# Congenital Left Atrial Appendage Aneurysm: The Role of Prenatal Fetal Echocardiography in Early Recognition

**DOI:** 10.7759/cureus.98614

**Published:** 2025-12-07

**Authors:** Omer A Algonaid, Rana S Alaskar, Omar Altamimi, Asma A Alsultan

**Affiliations:** 1 Pediatric Cardiology, King Fahad Medical City, Riyadh, SAU; 2 Pediatric Cardiology, King Fahad General Hospital, Riyadh, SAU

**Keywords:** atrial appendage aneurysm, atrial tachycardia, congenital cardiac surgery, fetal echocardiogram, systemic thromboembolism

## Abstract

Congenital left atrial appendage aneurysm (CLAAA) is a rare congenital cardiac anomaly that can lead to significant cardiac and systemic complications. Early recognition through fetal echocardiographic scanning is crucial for establishing a comprehensive management plan prior to delivery, including baseline postnatal assessment and close follow-up to allow timely surgical resection before the development of severe local or systemic complications such as heart failure or thromboembolism.

We report a case of giant CLAAA initially suspected as a fetal cardiac mass on routine obstetric ultrasound, which was subsequently characterized in detail by fetal echocardiography at our institution. The diagnosis was later confirmed postnatally by transthoracic echocardiography and computed tomography. When the patient developed atrial tachycardia, surgical resection of the aneurysm was performed successfully before the onset of any disabling systemic complications.

## Introduction

Congenital left atrial appendage aneurysm (CLAAA) was first described by Parmley in 1962 during a surgical procedure [1] and the diagnostic criteria’s where described as following: (1) origin from an otherwise normal left atrium (LA), (2) well-defined communication with the LA, (3) position within the pericardium, and (4) distortion of the left ventricle free wall by the aneurysm [2]. CLAAA has been reported in the literature worldwide, mainly through small retrospective studies or case reports, with variable descriptions [2-4]. One of these studies reached more than 80 cases [5]. The majority of these left atrial appendage aneurysm (LAAA) cases are asymptomatic. Nevertheless, symptoms like palpitations (43%), dyspnea (22%), and/or thromboembolic phenomenon (11%) are reported. Many factors were mentioned as a risk for systemic thromboembolism. However, only the tachyarrhythmia had a statistically significant association with such a complication [5].

Only two cases of giant CLAAA were reported in Saudi Arabia. The first case was a two-month-old with pneumonia, who also had an abnormal mass in the chest X-ray that blurred the left heart border and caused left lung collapse. These X-ray findings mandate further evaluation by echocardiography and computed tomography, which revealed the full diagnosis of LAAA with subsequent pathological confirmation after surgical resection [6]. The second case was a four-month-old, investigated for an incidentally detected murmur, where echocardiography diagnosed a giant LAAA that was confirmed by computed tomography scan [7].

## Case presentation

**Figure 1:**
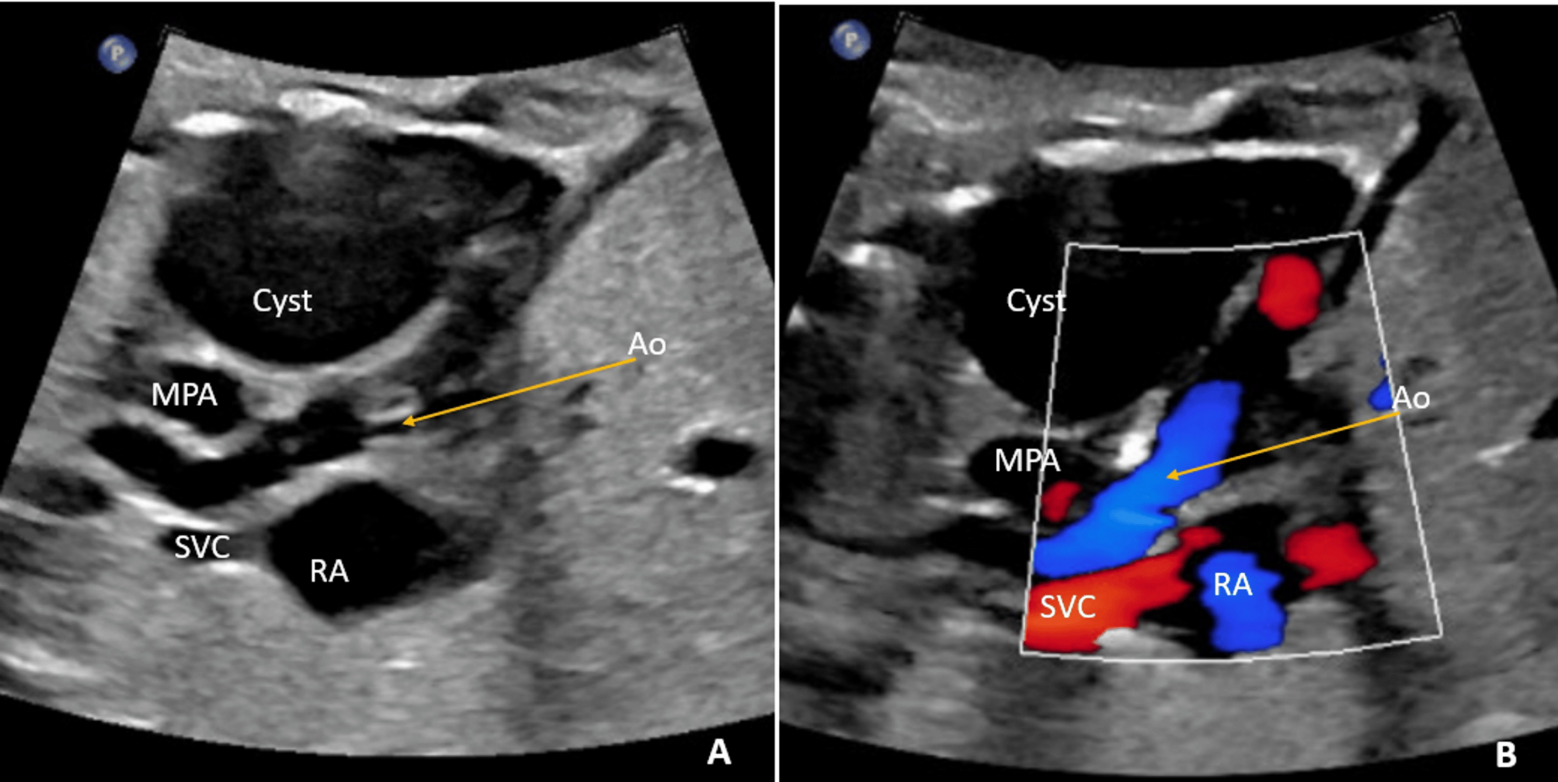
(A-B) Pre-natal ultrasound scan. A modified long-axis view of the three-vessel view showed the three vessels (SVC, aorta, and MPA) and a large single cystic mass adjacent to the left side of the fetal heart, but it failed to demonstrate communication with the LA. Ao: aortic valve; Cyst: congenital left atrial appendage aneurysm (CLAAA); MPA: main pulmonary artery; RA: right atrium; SVC: superior vena cava; LA: left atrium

A 32-year-old gravida 2, para 1 mother underwent a routine prenatal visit during which fetal ultrasonography revealed a single cystic mass adjacent to the left side of the heart, measuring 40 × 31 mm (Figures 1A-1B).

However, the color Doppler did not delineate the communication between the LA and this cystic lesion; therefore, the mother was referred for fetal echocardiography.

**Figure 2:**
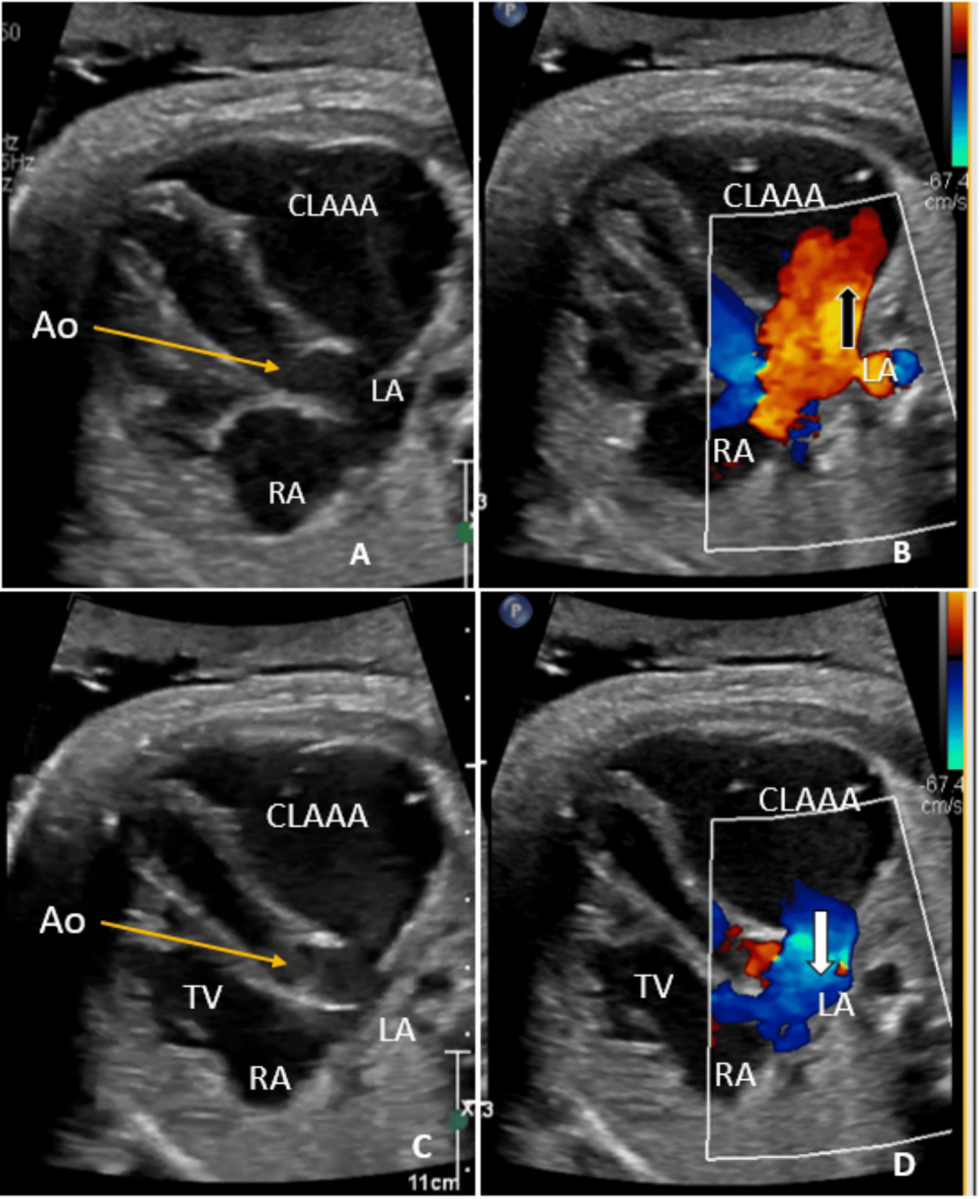
(A-D) Fetal echocardiography in a long-axis view, tilted cranially from the four-chamber view, showed a single cystic mass adjacent to the left side of the heart representing the CLAAA. Color Doppler demonstrated flow from the CLAAA to the LA (bold white arrow, image D) during diastole when the TV is open, and flow returning to it (bold black arrow, image B) during systole when the TV is closed, with forward flow seen across the Ao. The slim orange arrow indicates the level of the Ao. Ao: aortic valve; CLAAA: congenital left atrial appendage aneurysm; LA: left atrium; RA: right atrium; TV: tricuspid valve

The fetal echocardiographic assessment was performed twice, at the 37th and 40th weeks of gestation, which showed a large cystic structure posterolateral to the LA. This swelling was reported as a huge cystic-like structure adjacent to the LA and left ventricle, and it communicates with the LA, measuring 40 x 30 mm; such a description fulfilled the criteria of giant CLAAA. No fetal arrhythmias or intracystic thrombus were found (Figures 2A-2D).

The patient was delivered by assisted vaginal delivery (ventouse delivery). The initial clinical assessment showed a full-term baby boy, hemodynamically stable with no dysmorphic features.

**Figure 3:**
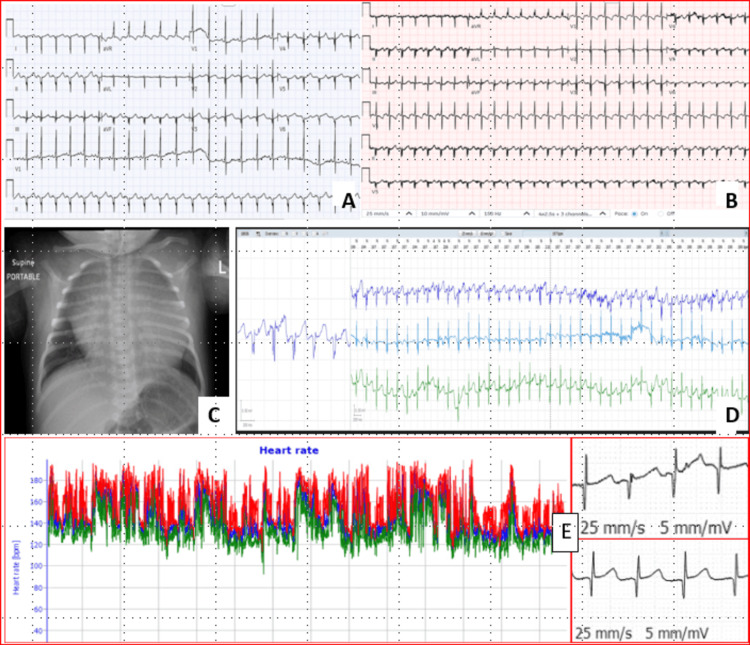
Overview of imaging and ECG findings. (A) Initial 12-lead electrocardiogram showed normal sinus rhythm with a heart rate of 137 beats per minute. There was a double-peak P wave with a normal axis. The QRS complex demonstrated a superior axis. (B) Follow-up 12-lead electrocardiogram showed an ill-defined P-wave morphology (negative P-wave axis in lead I, isoelectric in aVL, and positive in the other limb leads), suggestive of a left atrial origin, with a heart rate of 170 beats per minute and no obvious pre-excitation. There was right-sided dominance with extreme right-axis deviation and diffuse, nonspecific T-wave changes (likely a neonatal variant). (C) Anteroposterior chest X-ray in the supine position showed a globular cardiac silhouette with cardiomegaly and normal lung fields. (D) A good-quality 48-hour Holter monitor tracing showed a flat, fractionated P wave with 1:1 conduction and a short PR interval. (E) The Holter also demonstrated acceptable heart rate variability (52 milliseconds), with an average HR of 147 bpm, a minimum of 109 bpm, and a maximum of 193 bpm, along with an obvious change in the atrial focus during heart rate fluctuations, suggesting the presence of an additional non-sinus atrial origin.

The initial electrocardiogram (ECG) showed normal sinus rhythm with a rate of 140 beats per minute (Figure 3A), with occasional premature beats.

**Figure 4:**
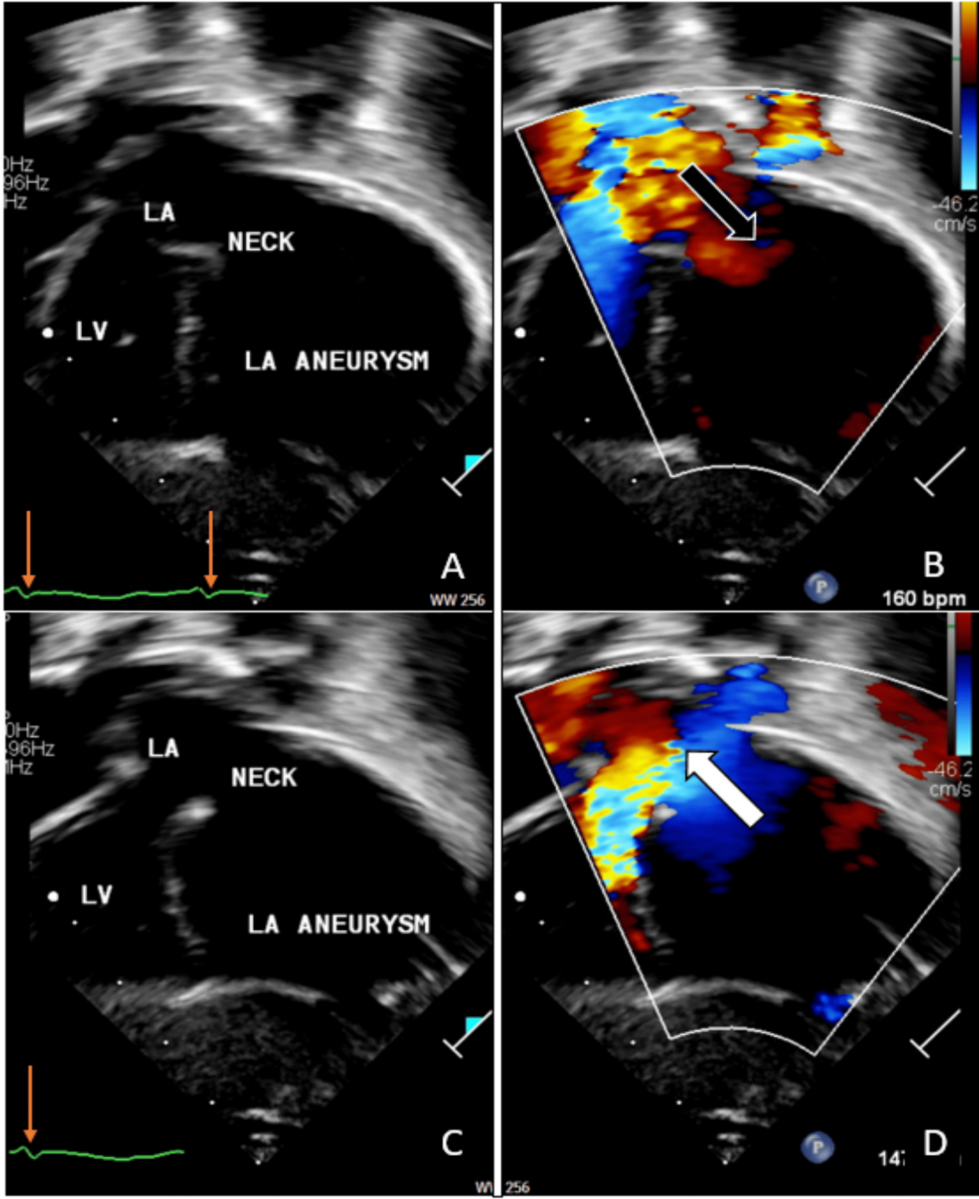
(A-D) Post-natal foreshortened modified apical transthoracic echocardiography showed a single cystic mass adjacent to the left side of the heart representing the CLAAA. Correlating with the ECG, Color Doppler demonstrated flow from the LA to the CLAAA (bold black arrow, image B) during systole, and flow returning from the CLAAA to the LA (bold white arrow, image D) during diastole. Slim orange arrows indicate a QRS. CLAAA: congenital left atrial appendage aneurysm; LA: left atrium; LV: left ventricle

Chest X-ray showed a globular heart with cardiomegaly and normal lungs (Figure 3C). The first post-natal transthoracic echocardiography (TTE) showed almost the same details as fetal echocardiography, which was reported as a huge cystic mass communicating with the LA and to-and-fro flow, most likely representing giant CLAAA, measuring 43 mm x 34 mm x 25 mm with about 6 mm communication orifice with LA, without intra-aneurysmal thrombus (Figures 4A-4D).

**Figure 5:**
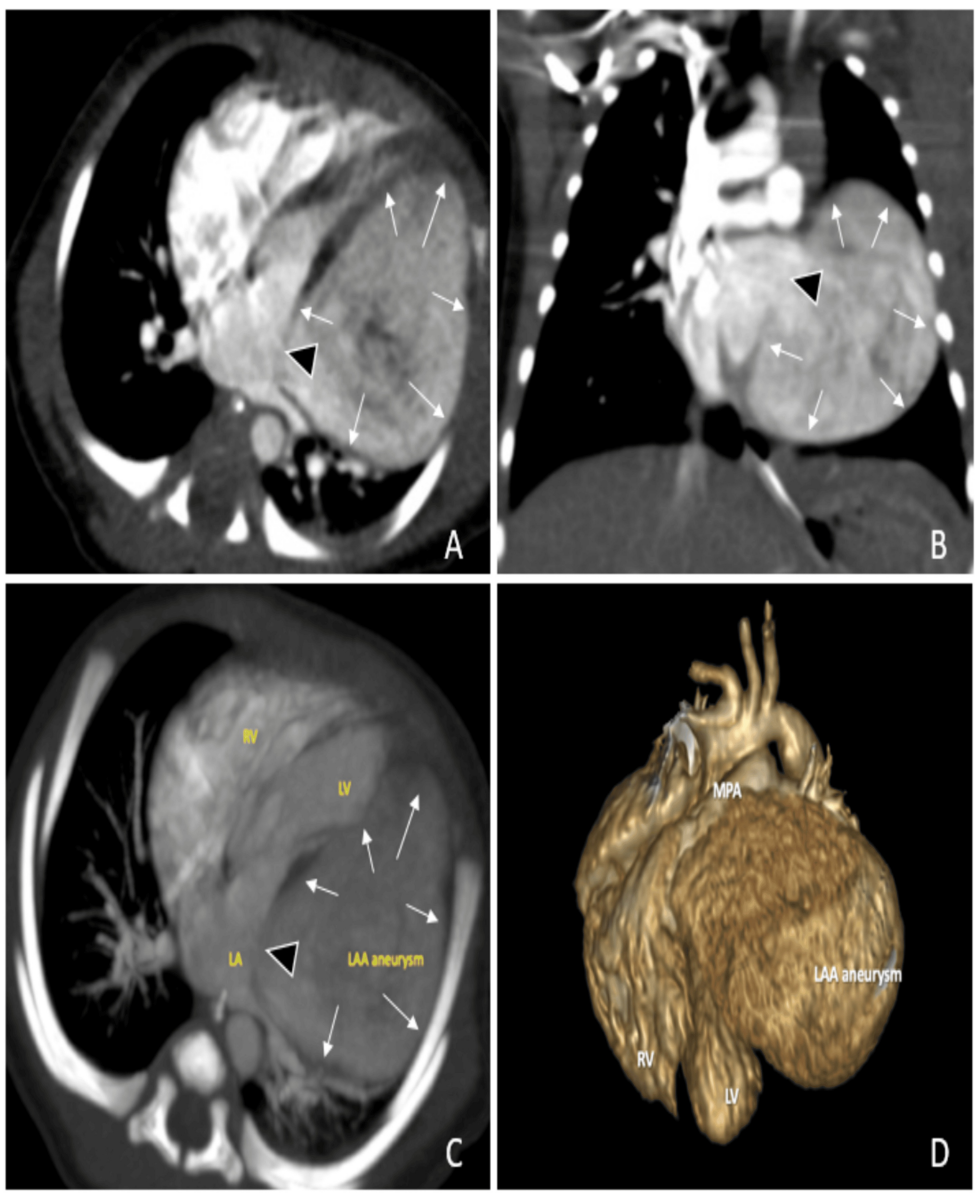
Computed tomographic angiography of the heart showed a single cystic mass adjacent to the left side of the heart. (A) Axial view, (B) coronal view, (C) maximum-intensity projection axial view, and (D) 3D reconstruction. LA: left atrium; LAA: left atrial appendage; LAAA: left atrial appendage aneurysm; LV: left ventricle; MPA: main pulmonary artery; RV: right ventricle; black arrow heads: communication of LAAA to LA (LAAA neck); small white arrows: extension of the LAAA

Initial laboratory investigations, including complete blood count, renal profile, electrolytes, and metabolic screening, are normal for age. Computed tomographic angiography (CTA) confirmed the echocardiography findings (Figure 5) and reported a massive dilatation of the left atrial appendage compressing the left ventricle. It communicates with the LA and measures about 46 x 45 x 36 mm. During hospitalization, no clinical concern was observed, specifically, no tachyarrhythmia by bedside ECG monitoring and 12-lead ECG. Nevertheless, the patient was kept on aspirin 5 mg/kg/day since diagnosis and then discharged home at three weeks.

In the first outpatient visit, at one month, TTE showed the same prior finding, but 12-lead ECG (Figure 3B) showed sinus tachycardia with a heart rate of 170 beats per minute, and 48-hour Holter monitoring showed additional non-sinus atrial rhythm (Figure 3D). No other arrhythmia was detected.

The patient was re-admitted for monitoring. Anti-arrhythmic medications were started, and the patient was prepared for surgical intervention. Due to family preference, however, the patient underwent surgery at another cardiac center in the area, where he was offered an aneurysmectomy; however, there was no documentation regarding a MAZE III procedure or pulmonary vein isolation.

## Discussion

The true incidence of CLAAA worldwide is unknown, and the incidence in Middle Eastern countries is also not established. Some retrospective studies report an incidence of 0.35% from fetuses screened by fetal echocardiography [8], and a more recent literature review published in 2024 revealed 177 cases [9] compared to an old one in 2013, which revealed only 82 cases [5]. The majority of these patients are asymptomatic and detected incidentally at different ages during either irrelevant diagnostic procedures or retrospectively by reviewing the patient's old diagnostic workup that was saved in the hospital archives as part of a research project [5]. The remaining smaller group was diagnosed after the appearance of symptoms of advanced local effects [10,11] or systemic complications [12], which makes early diagnosis crucial to prevent such a complicated presentation. Rarely, LAAA has been reported to be associated with congenital cardiac abnormalities - atrial septal defect, ventricular septal defect, anomalous pulmonary venous drainage, mitral valve cleft, and tricuspid atresia [13]. However, this patient has no associated congenital heart anomalies.

Early detection of the asymptomatic CLAAA helps the treating team offer these patients proper intervention while the patients are not yet symptomatic, either at the time of appearing a risk factor for a local or systemic complication like arrhythmias or at least at the earliest manifestation [5]. One of the earliest diagnostic tools is fetal ultrasound scanning, and more accurate is fetal echocardiography [8]. In our reported case, the fetal echocardiography was able to describe the full diagnostic criteria of CLAAA [2] with great accuracy and was confirmed by neonatal TTE; however, due to the rarity of this anomaly, we proceeded to CTA. These neonatal evaluations still have another valid indication, including the follow-up for any evolving complications like thrombosis, especially before any intervention or to observe for any significant increase in size, especially with the manifestation of local effects like compression of the airway and lungs [10] or coronaries [11].

There are a few non-surgical treatments for CLAAA. The aims of medical treatments are mainly to treat the associated findings, like tachyarrhythmia and thromboembolism. The AtriClip is a device that was recently introduced for use in occluded LAAs, but incomplete closure by this device has been frequently reported [14]. They reported that a residual stump occurred in three (13%) of 24 patients who underwent LAA closure with AtriClip [14]. Even though the above-mentioned options are still valid in many cases, the definitive treatment remains the surgery [15].

Another conflicting issue in the current literature is the time of surgical intervention, as there is a recommendation for resection immediately after diagnosis, regardless of the manifestation [5]. In contrast, others suggest resecting the CLAAA if the risk factors of a complicated course occur, like tachyarrhythmia [12].

Surgical resection of LAAA in asymptomatic patients is recommended [15], although some debate remains, as about 24% continue to be asymptomatic [9], and there are reports of incidentally diagnosed adult cases doing well with conservative management [16]. However, we thought that there is no rationale to wait for devastating local or systemic complications in the presence of the risk factors, especially tachyarrhythmias [10,12].

The type of surgical procedure - aneurysmectomy versus total appendectomy - and the approach - sternotomy versus lateral thoracotomy - are other conflicting issues, where we need to weigh the benefit of preserving appendage function against avoiding the unproven risk of aneurysm recurrence and future potential anatomic source of thromboembolism [15,17,18]. Although resection alone is usually adequate, in some cases, especially with bi-atrial enlargement, an additional Cox-Maze III procedure or pulmonary vein isolation is performed [19].

## Conclusions

CLAAA is a rare anomaly. The early diagnosis can help to establish a proper management plan to avoid potential local or systemic complications. We report a case of a giant CLAAA that was completely diagnosed at our institution by fetal echocardiography and surgically resected at the age of two months, prior to the onset of any systemic complications and in view of the known risk of tachyarrhythmia. Regular screening is recommended to identify residual channels, and complete endocardial closure of an incompletely ligated LAA may be a reasonable option for patients who cannot tolerate long-term anticoagulation therapy.
